# Delayed neurological deterioration after surgery for intraspinal meningiomas: Ischemia-reperfusion injury in a rat model

**DOI:** 10.3892/ol.2015.3626

**Published:** 2015-08-19

**Authors:** LIANG WU, TAO YANG, CHENLONG YANG, NING YAO, HUILIANG WANG, JINGYI FANG, YULUN XU

**Affiliations:** 1 Department of Neurosurgery, China National Clinical Research Center for Neurological Diseases, Beijing Tiantan Hospital, Capital Medical University, Beijing 100050, P.R. China; 2 Department of Physiology and Pharmacology, Karolinska Institute, Stockholm 11251, Sweden; 3 College of Chemistry, Beijing Normal University, Beijing 100875, P.R. China; 4 Department of Neuropathology, Beijing Neurosurgical Institute, Capital Medical University, Beijing 100050, P.R. China

**Keywords:** ischemia-reperfusion injury, intraspinal meningioma, chronic compression, neurological deterioration, postoperative complication

## Abstract

Delayed neurological deterioration in the absence of direct cord insult following surgical removal and cord decompression is a rare but severe postoperative complication in a small subset of patients with intraspinal meningiomas. To date, the underlying pathophysiology of such a finding remains unclear and ischemia-reperfusion injury (IRI) is considered as the potential etiology in the literature. However, no experimental research has been reported to prove this hypothesis. The present study investigated whether IRI occurs following decompression surgery using an experimental rat model of chronic compressive spinal cord injury (SCI). A chronic spinal cord compression model was developed with various sizes of polymer sheets (mild and severe compression) that were microsurgically implanted underneath the T8-9 laminae, and occurrence of IRI in the spinal cord following decompression was determined by measuring superoxide dismutase (SOD) level and malondialdehyde (MDA) concentration. In the mild compression groups, after decompression SOD activities significantly increased along with a reduction in MDA content compared with the non-decompression group (P<0.05), which exhibited diminishment of lipid peroxidation and relief of the secondary injury. These findings indicated that decompression is effective to improve neurological recovery and may deliver improved outcomes for chronic mild compression of the spinal cord. However, in severe compression groups, after decompression, SOD activities markedly reduced further along with a significant increase in MDA content compared with non-decompression group (P<0.05). The results indicated that lipid peroxidation increased immediately after decompression surgery which resulted from reperfusion of the spinal cord. These findings demonstrated IRI may occur as a result of chronic severe compression of the spinal cord. In clinical practice, sudden cord expansion and reperfusion may have lead to disruption in the blood spinal cord barrier, and triggered a cascade of IRI resulting in postoperative neurologic deterioration. Recognition of this neurological deterioration following removal for intraspinal meningiomas may improve preoperative patient counseling and merits further study for determination of the precise pathophysiology.

## Introduction

Meningiomas are common tumors that occur within the spinal canal. Since chronic compression to the spinal cord is the main pathological mechanism, complete removal of the meningioma for cord decompression is the primary treatment choice. With development of modern neuroradiological techniques and standard microneurosurgical procedures, surgical treatment is usually successful with low morbidity and good outcomes (1–3). However, delayed neurological deficit in the absence of direct cord insult following surgical decompression often occurs in patients with chronic compressive spinal disorders, including cervical spondylotic myelopathy, ossification of the spinal ligament, and spinal stenosis. Delayed neurological deficit is also a rare but severe postoperative complication observed in a small subset of patients with intraspinal meningiomas (4–8).

A previous clinical study reported on 284 patients who received surgery for a spinal meningioma at Beijing Tiantan Hospital (Beijing, China) between the years 2004 and 2010 (9). A total of 10 patients exhibited delayed but severe neurological deterioration following complete removal of their tumors in the absence of any direct trauma to the cord. Of these patients, there were 5 male and 5 female patients with a mean age of 46.8 years. The mean duration of illness from onset of symptoms to diagnosis was 42.8 months. Seven tumors were located in the thoracic spine and 3 in the cervical spine. The tumors compressed the cord severely and gross total removal was achieved in all the cases. Immediately after the surgery, all the patients could move all their extremities but the onset of the neurologic deterioration became apparent during the 3–8 h following surgery in all the cases (mean 5 h). To date, the underlying pathophysiology of these findings remains unclear and ischemia-reperfusion injury (IRI) is considered as the potential cause in the literature (4,6,8). Moreover, since all 10 patients suffered severe cord compression, the degree of compression may be a risk factor of IRI. However, to the best of our knowledge, no experimental research has been reported to prove this theory of etiology.

The present study investigated whether IRI occurs following decompression surgery using an experimental rat model of chronic compressive spinal cord injury (SCI). Lipid peroxidation reaction resulting from oxygen-derived free radical overproduction initiates following IRI and is one of the main pathological mechanisms (10,11). Therefore, the levels of superoxide dismutase (SOD) and malondialdehyde (MDA) prior to and following decompression were biochemically measured and analyzed, both of which are important and reliable markers of lipid peroxidation reaction (12,13), to determine the occurrence and extent of IRI. To the best of our knowledge, this is the first reported experimental study that investigates the underlying causes of atraumatic neurological deterioration following surgery for intraspinal meningiomas.

## Materials and methods

### 

#### Animal and experimental groups

All the animal experiments were approved by the ethics committee of Beijing Tiantan Hospital, Capital Medical University, and performed in accordance with the policies of Chinese animal research committees and guidelines from U.S. National Institute of Health (NIH publication No. 96-23, revised 1996). Sprague-Dawley (SD) rats were provided by the Experimental Animal Facilities of the hospital. The number of animals used and their suffering were minimized.

Thirty male rats (280–320 g) were randomly assigned into 6 groups. Detailed information about groups is presented in Table I. The rats in each group were kept in separate cages in rooms with controlled light and temperature and were fed standard chow and water ad libitum. Room temperature was set at 25±3°C.

#### Rat model of chronic compressive SCI

Rat model of chronic compressive SCI was established in accordance with the model of Wang *et al* (14) and Kim *et al* (15). All animals were prevented from drinking in the morning of surgery. Animals were anesthetized by an intraperitoneal (i.p.) injection of trichloroacetaldehyde (300 mg/kg; Qingdao Yulong Algae Ltd., Qingdao, China; No. H37022673) and placed on a thermistor-controlled heating pad in prostrate position. The fur of the animals was shaved around chest and abdomen. Following disinfection, spinous processes and laminar arcs of T7-10 were exposed following T5-12 midline skin incision and paravertebral muscle dissection. The yellow ligament between the laminae was removed and the dura underneath was separated from the laminae carefully so as to not result in a cerebrospinal fluid leak. The expanding compression material (mild compression size: 2.5 × 2.0 × 0.4 mm^3^; severe compression size: 2.5 × 2.0 × 0.8 mm^3^) was inserted between the T8 and T9 laminae and dura. For the sham group, the protocol was the same as for the experimental group, except for the insertion of the compression material.

The compression sheet was made of a water-absorbing material, which is a penetrating polymer network hydrogel composed of polyvinyl alcohol and polyacrylamide (1:1). The surface of the hydrogel is crosslinked with glutaraldehyde 10 times, and the surface has long-term water retention capabilities. After absorbing water, the expansion of the materials is mainly reflected as an increase of its thickness and the final volume remains stable for a long period of time without decomposition. The expansion rate can gradually reach a maximum of 3 times its original thickness (mild compression size: 3.0 × 2.5 × 1.2 mm^3^; severe compression size: 3.5 × 3.0 × 2.4 mm^3^). Prior to the experiment, the material was implanted subcutaneously in the abdomen of rats. After 3 months, no obvious inflammation or other abnormal tissue reactions were observed using histological analysis.

Surgical procedures were performed under sterile conditions with the assistance of a surgical microscope. Bleeding control was performed with a bipolar coagulator. Subsequently, the muscle and skin were sutured in layers with 6-0 Vicryl (Ethicon, Johnson & Johnson Intl, Lanneke Marelaan, Belgium). Following the surgical procedure, the rats were placed in a warming chamber and their body temperatures were maintained at ~37°C until they were completely awake. Ampicillin liquid formulation (80 mg/kg; Suzhou Two Leaves Pharmaceuticals Inc. Ltd., Suzhou, China, Batch No. H32021320) was injected into the back exterior muscles once per day for 3 days to prevent infection. Temperatures were strictly maintained and all the rats were housed individually with free access to food and water. Padding in each cage was changed every day to keep it dry. After surgery, bladder massage was performed twice per day to stimulate autonomic urinary reflex.

Rats were sacrificed 12 weeks following surgery. In the decompression groups, rats underwent laminectomy and removal of the expanded materials and the animals were kept alive for 24 h after decompression surgery under appropriate conditions and veterinary control, after which decapitation took place after anesthetization using the same anesthetic agents. In the sham-d group, rats underwent laminectomy and the protocol after surgery was the same as the experimental group. Spinal cord samples (15 mm) were obtained from the compressed spinal cord area and divided into two equal parts. Cranial parts of the tissue samples were obtained for microscopy evaluation; caudal parts were cleaned of blood with a scalpel and immediately stored in a −20°C freezer for biochemical analysis.

#### Evaluations of animal model

All magnetic resonance imaging (MRI) experiments were conducted with a 3 Tesla MRI (Magnetom Trio, Siemens Medical Solutions, Erlangen, Germany). T2-weighted images (Echedelay time=92 ms; Repetition time=3620 ms; flip angle α=120°; slice thickness: 2 mm; Field of view=80 mm) were obtained at the 12th week following the operation. All rats were placed in a prone position. Images of the thoracic spinal cord were acquired in the axial and sagittal planes. The cross-sectional area was measured using the Siemens NUMARIS system software, version 4 (Siemans Healthcare, Erlangen, Germany).

Neural function was scored to evaluate the animal model of chronic compressive SCI in an open field according to the Basso, Beattie and Bresnahan (BBB), locomotor rating scale of 0 (complete paralysis) to 21 (normal locomotion) (16). BBB scores categorize combinations of rat hindlimb movements, joint movement, weight support, fore/hindlimb coordination, trunk position and stability, stepping, paw placement, toe clearance, and tail position, representing the sequential injury stages that rats take after SCI. Rats are permitted to move freely and scored over 4 min by 2 independent observers. Locomotion activity of the hindlimb was evaluated once per week following the surgery until the time of sacrifice. The ranking standards were established as follows: i) The activity of hindlimb joints were scored between 0 and 7; ii) the pace and coordination of the hindlimbs were evaluated; and iii) the fine activities of paws during locomotion were evaluated. Each evaluation was completed by two independent observers who were blinded to the experiments and the values were represented as the mean ± standard deviation.

#### HE staining and Nissl staining

The specimen was immersed into 4% paraformaldehyde in 1X phosphate buffered saline (4% PFA) and stored at 4°C for post-fixation. One week after the fixation, they were removed from the store and placed in fresh fixative. Fixed tissue samples were processed routinely by paraffin embedding technique following dehydration. A total of 150 serial cross sections with thickness of 5 µm were obtained from each rat, and processed with hematoxylin and eosin staining (HE) and Nissl staining (Boster Biotechnology, Wuhan, China). Motor neurons were identified by the presence of large nuclei with well-developed, densely staining Nissl bodies in the cytoplasm (14). In addition, the nucleus, which is typically located centrally in the cell, contains a well-demarcated round nucleolus. To determined the density of large-sized neurons in the gray matter and obtain a precise stereologicount of the neurons, a slice thickness of 5 µm and a gap interval of >8 µm were selected, based on the following stereological considerations. The characteristic large nucleoli have a fairly uniform diameter of ~5 µm (13,14). Selecting a slice thickness identical to the diameter of the spherical nucleolus means that each section will contain tangential contours of the nucleoli with their centers located within 2.5 µm outside of the slice. Thus, counting nucleoli of the motor neurons that appear on a 5-µm slice yields the number of those with the center located within 5 µm on each side from the middle of the slice. Leaving >8 µm intervals between the sections means the stereological count can avoid omission or redundancy in the number of motor neurons. Stereological counting of neurones in the preparations was performed using a light microscope (CH30; Olympus, Tokyo, Japan). All the sections were evaluated morphologically by the same pathologist who was blinded to each group to assess the histopathological change.

#### Biochemical analysis

Tissues from each group were homogenized at a concentration of 100 g/l after cutting the organs into small pieces, centrifuged at 5,000 × g for 20 min at −10°C, and stored at −20°C. Biochemical kits (Jiancheng Institute of Biology, Nanjing, China) were used to measure levels of SOD and MDA, in accordance with the protocol. The activity of SOD was expressed as units per mg protein. One unit of SOD was defined as the amount of protein that inhibited the rate of nitroblue tetrazolium reduction by 50%. The levels of the end product of lipid peroxidation, tissue MDA, were expressed as nmol/mg.

#### Statistical analysis

SPSS software, version 16.0 (SPSS, Inc., Chicago, IL, USA) was used for statistical calculations and graphs. Data are expressed as the mean ± standard deviation. Samples from each group were compared using one-way analysis of variance. P<0.05 was considered to indicate a statistically significant difference.

## Results

Postoperatively, rats were in a good, healthy condition and did not develop any infections. Depending on the degree of compression, a graded outcome was evident from radiography, neurological tests, and light microscopic examination as is described below.

### 

#### Neuroradiological observations

**Figure 1.**
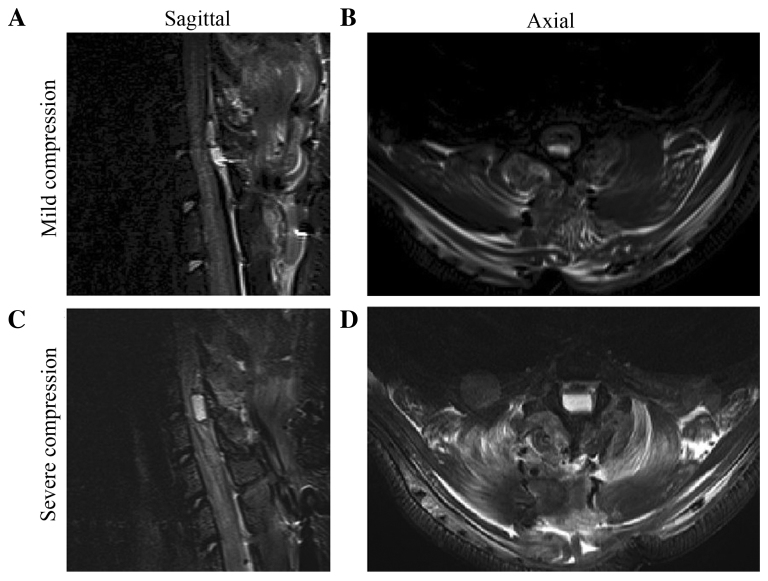
Magnetic resonance images in mild and severe compression groups. T8-9 levels allowed determination of mild compression in (A) sagittal and (B) axial T2-weighted images; and severe compression in (C) sagittal and (D) axial T2-weighted images. The sheet was located dorsally within the spinal canal and compressed the cord obviously by different degrees.

The spinal cords were progressively compressed. Sagittal and axial projections of the thoracic spine were obtained to ascertain the location of the compression sheet and to evaluate the degree of spinal cord compression (Fig. 1). The cross-sectional area in the sham group was larger than in the compressed groups and a significant difference was observed between the MC and SC groups (P<0.05) (sham: 16.39±1.42 mm^2^; MC: 11.24±2.87 mm^2^; SC: 4.31±1.59 mm^2^).

#### Neurobehavioral outcomes

**Figure 2.**
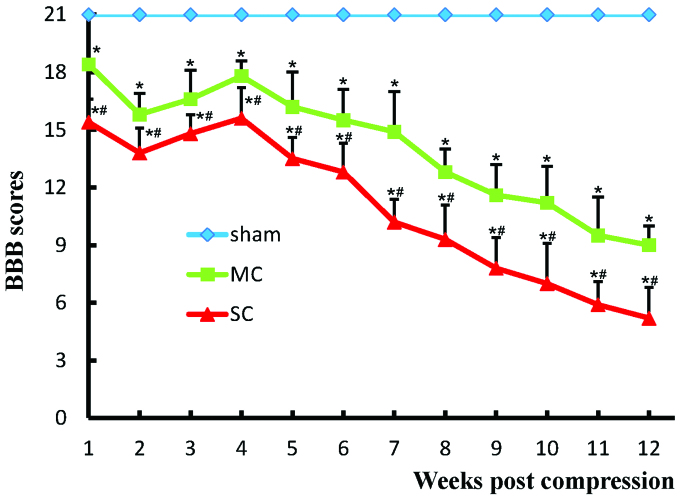
Neurobehavioral changes following spinal cord compression. Compared with the sham group, progressive neural function impairment were detected in the compression groups with significant difference between the mild versus the severe compression group. *P<0.05 vs. sham group; ^#^P<0.05 vs. mild compression group.

All rats in the sham and sham-d group had a normal postoperative neurological outcome (BBB score of 21). Rats with compression had graded neural function injury depending on the time and degree of compression (Fig. 2). In the MC group, rats suffered from progressively mild motor injury. In SC group, rats demonstrated severe motor injury and all were observed to have paralysis, which was indicated by markedly reduced BBB scores. A significant difference was detected between the sham group and the compression groups (P<0.05). The BBB scores in the MC and SC groups at all timepoints post-surgery were statistically significant different (P<0.05).

#### Histopathological observations

**Figure 3.**
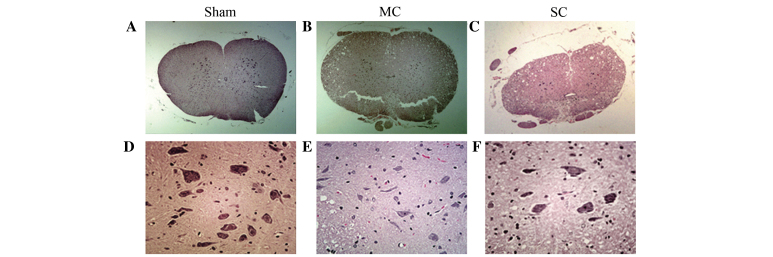
Hematoxylin and eosin (HE) staining of the spinal cord samples (original magnification, A-C: x4; D-F: x40). (A and D) HE staining showed normal neural and glial morphology in the sham group. (B and E) In the mild compression (MC) group, neurons displayed normal morphology with clear boundary. Compared with the severe compression (SC) group, mild glial proliferation, hemorrhage and edema occurred in the MC group. (C and F) In the SC group, impacted spinal cord exhibited typical necrosis showing as broad hemorrhage, edema, reactive gliosis and neural apoptosis with condensed nuclei.

Histopathological alterations in the spinal cord after injury were also compared. HE and Nissl staining were used to analyze histopathological change. HE staining demonstrated that the spinal cord in the sham group had integrated infrastructures and clear boundary between gray and white matters, the blood vessels and central canal also exhibited normal morphology (Fig. 3). In addition, no neuronal apoptosis and glial proliferation were observed in the sham group (Fig. 3). Instead, in compression groups the spinal cords were observed to have progressive pathological changes and the extent of compression damage was proportional to the neurological score. The number of neurons in the gray matter of chronically compressed spinal cords reduced progressively with the increase in degree of compression. In the MC group, a portion of neurons were observed with condensed nucleus, darkly red stained cytoplasm and also appearance of apoptotic bodies. However, a proportion of neurons remained which indicated blurring structures. In the SC group, the spinal cord was notably flattened at the site of compression. In the gray matter, neurons were flattened, small, and reduced in number and patches of necrosis were seen. Chromatolysis, both central and peripheral, was observed in the remaining neurons. In the white matter, graded myelin damage and loss of axons and glia were noted, as was status spongiosis. Cavity formation and myelin ovoids were observed in the anterior, lateral, and posterior columns. Myelin ovoids may have resulted from phagocytosis of degenerating axons and the myelin sheath. The spinal cord lacked clear infrastructures and cellular boundaries compared with the MC group. Vessel stenosis and occlusion in the gray and white matter became progressively worse with the increasing of degree of compression, which demonstrated the development of spinal cord ischemia.

**Figure 4.**
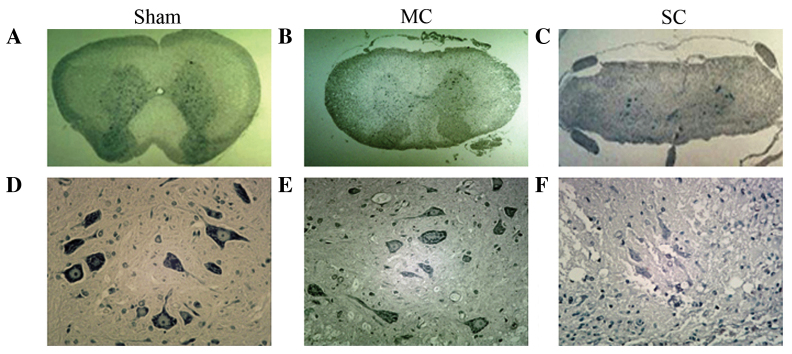
Nissl staining of the spinal cord samples (original magnification, A-C: x4; D-F: x40). (A and D) In the sham group, neurons exhibited a large amount of granule like and densely stained toluidine blue in the cytoplasm. However, (B and E) in the mild compression group, the number of Nissl bodies decreased and displayed patch morphology. (C and F) In the severe compression group, the Nissl bodies dramatically decreased or even disappeared in neurons compared with that of the mild compression group.

Nissl staining demonstrated that neurons in the sham group displayed integrative and granular-like morphology (Fig. 4), and the number of motor neurons was 1050.5±128.2. The plasma was densely stained with toluidine blue, indicating an active supply of neuronal nutrients and energy synthesis. In the MC group, retained neurons were observed, although the number was reduced (582.0±69.5) and tissue morphology was relevantly maintained with lighter staining in the cytoplasm and granular-like morphology. However, in the SC group the number of neurons was notably reduced (274.6±92.4) and neurons appeared irregular morphologies. Intracellular toluidine blue staining was also significantly reduced, dimly spread out and apparent in the periphery of the cytoplasm, which indicated chronic compressive SCI-induced neuronal necrosis and apoptosis lead to neuronal loss. The remaining neurons may have difficulties in energy synthesis resulting in neural dysfunction.

**Figure 5.**
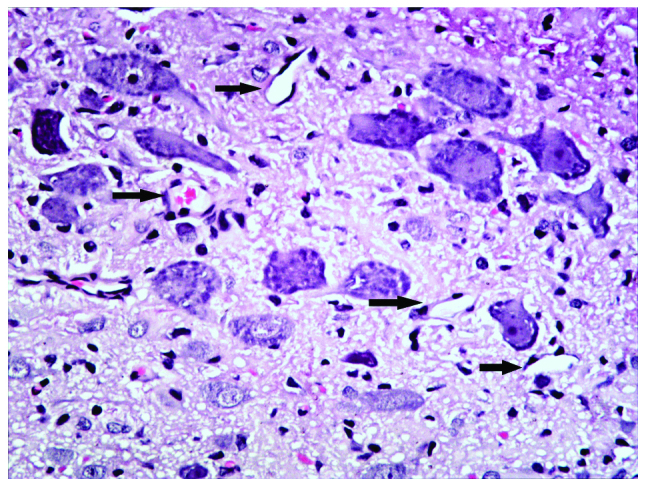
Dilated blood vessels (*black arrow*) were observed in the chronic compressive spinal cord after removal of the expanded sheets for decompression.

No histopathological changes were observed in the sham-d group. In the decompression groups, histopathological manifestations exhibited no obvious changes compared with those in the compression groups to the same degree and histopathological improvement the in spinal cord was not apparent because it was observed at a very early stage following decompression surgery and the spinal cord had not had enough time to recover. However, blood vessels in both gray and white matter were markedly dilated (Fig. 5).

#### Levels of SOD and MDA

**Figure 6.**
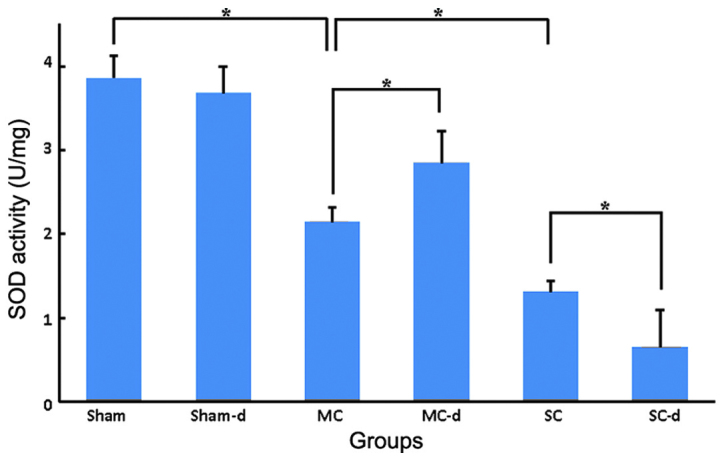
Superoxide dismutase (SOD) activity of the spinal cord tissues. Compared to the sham group, SOD activities in the compression group significantly decreased and they were lower in the severe compression (SC) group than those in the mild compression (MC) group. After decompression (d), SOD activities in MC-d group significantly increased compared to the MC group. However, the activities in SC-d group declined further compared to SC group. *P<0.05.

**Figure 7.**
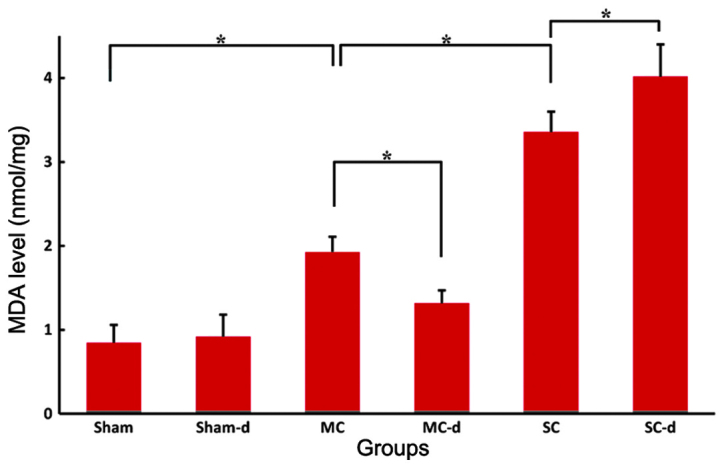
Malondialdehyde (MDA) level of the spinal cord tissues. Compared to the sham group, MDA level in the compression group significantly increased and it was higher in the severe compression (SC) group than that in the mild compression (MC) group. After decompression (d), MDA levels in MC-d group significantly decreased compared to the MC group. However, the levels in SC-d group increased more compared with the SC group. *P<0.05.

The SOD activity of the spinal cord tissues from the sham group was 3.86±0.27 U/mg (Fig. 6) and the MDA level was 0.85±0.21 nmol/mg (Fig. 7). In the compression groups, chronic compressive SCI resulted in a significant graded reduction in SOD activity (MC: 2.14±0.18 U/mg, SC: 1.31±0.13 U/mg, P<0.05) and increase in MDA level (MC: 1.93±0.18 nmol/mg, SC: 3.36±0.24 nmol/mg, P<0.05). No significant differences were observed in the sham-d group compared with the sham group: SOD activity of the spinal cord tissues was 3.68±0.32 U/mg and MDA level was 0.92±0.26 nmol/mg. Compared with the compression group, decompression surgery following removal of the expanded materials in the MC-d group markedly rescued the SOD activity to 2.85±0.38 U/mg (P<0.05) and significantly reduced the MDA level to 1.32±0.15 nmol/mg (P<0.05). Instead, in the SC-d group SOD activity was significantly reduced to 0.65±0.44 U/mg (P<0.05) and MDA level was elevated higher to 4.02±0.38 nmol/mg (P<0.05) in comparison with those of the SC group rats.

## Discussion

A previous study reported on the outcomes of 284 patients who received surgery for intraspinal meningioma at Beijing Tiantan Hospital and summarized the clinical features of 10 patients presented with delayed neurological deterioration postoperatively with unknown cause. It has been proposed that patients and surgeons should be aware of the potentially catastrophic results after a seemingly routine tumor removal to treat an intraspinal meningioma with chronic but severe cord compression. It is necessary to explain the rate of neurologic deterioration and possible complications that may arise following surgery and to do this prior to surgical intervention. In general, the postoperative neurological deficit is most often due to mechanical damage of surgical procedures and intraspinal hematoma (17–19). However, careful surgical technique and intraoperative neuromonitoring may indicate any potential trauma to neural tissue during tumor removal and decompression procedure. In the absence of clear etiology, spinal cord IRI is considered to be responsible in previous studies (8,10,20). Microcirculatory disturbance due to reperfusion may occur in any level and any location where surgical decompression was performed for the chronic compressive lesion (8,21). However, no previous studies have proven this theory in this rare postoperative complication. In the present study, an experimental rat model of chronic compressive SCI was established with or without decompression surgery to identify whether spinal cord IRI is the potential etiology of patients with unknown postoperative neurological deterioration.

There have been previous experimental animal models of chronic spinal cord compression, including the placement of screws and subsequent gradual tightening of the screws, the epidural transplantation of tumor cells in rats, and the epidural implantation of expanding materials (14,15,22–28). Numerous investigators have used twy mice, which are a model of spinal ligament ossification (29–32). In the current study, a chronic spinal cord compression model was produced using water absorbing materials to imitate the compression process and the animal model was evaluated using neuroradiology, neurobehavior, and histopathology.

In the compression groups, MRI performed at the schedule time demonstrated that the spinal cord was notably compressed and the mean spinal cord narrowing rate was significantly different between the MC and SC groups, which indicates different effects of graded compression in the animal model. Assessment of neurologic function is a prevalent method for accessing the degree of neural injury. In the present study, compared with sham group, the BBB rating demonstrated a progressively significant deterioration of locomotion in compression groups at all tested time points post-surgery. Moreover, behavioral scores were significantly different between MC and SC groups at every timepoint. Due to the powerful spinal cord self-repair mechanisms in rats, the behavioral function of rats following compression gradually recovered in the early phase (3–4 weeks post-surgery) of the experiment. However, the animal models in the present study were evaluated in the late phase (after 12 weeks of compression) and the effect of the self-repair was very limited. Therefore, implantation of the expandable sheets in the present study reduced the locomotive function in rats and may have simulates the behavioral changes of chronic compressive SCI.

The spinal cord is morphologically similar to a cylinder where gray matter is surrounded by white matter (33). Compared to white matter, gray matter has a low density with loosely connected tissue and is full of blood vessels. Histopathologically, when the spinal cord is compressed from behind, more cells are dislodged and the damage is more serious in gray matter than that in white matter (27,28). In the present study, mild edema and ischemia, reactive gliosis and neuronal apoptosis with condensed nuclei were observed in the MC group. However, in the SC group a significant deterioration in the above abnormal ultrastructures was observed, indicating that implantation of the expandable sheets may gradually worsen the neurological function of the animal model through progressing tissue structure pathological changes. Furthermore, Nissl staining was used to identify whether spinal cord neurons underwent pathological changes. Nissl bodies are large granules observed in neurons, which may be identified by Nissl staining and are often used to demonstrate the neural structure of the spinal cord. Nissl bodies are actually the rough endoplasmic reticulum (with ribosomes) and are the site of protein synthesis, consisting of important constituent which is associated with the nutritional condition of neurons (34). Therefore, a large amount of large Nissl bodies may indicate neurons with dynamic protein synthesis and energy supply. Nissl bodies exhibit changes under various physiological conditions and in pathological conditions they may reduce in number, dissolve and even disappear (13,28). In the present study, the spinal cord tissues were observed with Nissl staining. In the rats with chronic mild compression injury, lighter Nissl staining in the cytoplasm and granular-like morphology in neurons was observed, indicating neuronal function was retained. This was further demonstrated by a restored neuronal number in the spinal cord in the animals with mild compression injury. Whereas a significant reduction in Nissl staining and a marked reduction in the number of neurons was observed in the rats with severe compression injury, indicating a loss of neurons due to the necrosis and apoptosis. The number of surviving neurons in the MC group (582.0±69.5) was significantly increased compared with that in the SC group (274.6±92.4; P<0.05); however, it was significantly reduced compared with the sham group (1050.5±128.2; P<0.05). In the decompression groups, the blood vessels were notably dilated, which indicated that the blood supply in the spinal cord was partially restored and the ischemia condition of neural tissues may be relatively relieved. No other obvious histopathological changes were observed in the spinal cord since they were observed at the very early stages following decompression surgery. The spinal cord of rats had limited time to exhibit a response to decompression and therefore remained relatively stable at cellular levels. Taken together, the present animal model may be considered to be useful for future studies on chronic compressive SCI.

In the present study, the occurrence of IRI in the spinal cord after decompression was examined by measuring the SOD level and MDA concentration. SOD is an enzyme that catalyzes the dismutation of superoxide anions. It is a major intracellular anti-oxidative enzyme that scavenges free radicals to protect cells from oxidative damages (35,36). The level of SOD represents the ability of tissues and cells to evade the toxicities of free radicals. Metabolic bursts, in which oxygen is reduced to superoxide (O_2_^−^), hydrogen peroxide (H_2_O_2_), and hydroxyl radical, may be elicited by various stimuli (37). SOD eliminates superoxides by converting them to H_2_O_2_. H_2_O_2_ is finally reduced to water by cytosolic antioxidants, catalase (CAT), and glutathione peroxidase (GSH-Px) (38). MDA is the breakdown product of the major chain reactions leading to the oxidation of polyunsaturated fatty acids and may determine the extent of the peroxidation reaction (35). It has previously been established that reperfusion of neural tissue may have deleterious clinical sequelae likely associated with the role of reactive oxygen radical-mediated neuronal cell death (39). Animal models have demonstrated that superoxide-mediated injury immediately occurs following reperfusion during neuronal ischemic events (35–37). Therefore, SOD and MDA frequently serve as important and reliable markers of oxidative stress-mediated lipid peroxidation that reflect the current reperfusion injury status following spinal cord decompression.

Following chronic compressive SCI, in addition to the direct damage at injured location, lipid peroxidation due to free radical overproduction is the main pathological change of the secondary injury that starts after chronic SCI. The results of the present study demonstrated that compared with the sham group, SOD activities in the compression groups significantly reduced, along with a notable increase in MDA concentration. Moreover, there was a reduced levels of SOD activity and an increased concenttration of MDA in the SC group compared with the MC group, which indicated progression of the secondary injury had occurred. Following decompression, SOD activities in the MC-d group significantly increased along with a reduction in MDA contentration compared with the MC group, which demonstrated diminishment of lipid peroxidation and relief of the secondary injury. These findings indicate that decompression is effective to improve neurological recovery and may deliver improved results for chronic mild compression of the spinal cord. However, SOD activities in the SC-d group declined further along with a dramatical increase in MDA content compared with the SC group. The results reflected lipid peroxidation increased immediately following decompression surgery which was resulted from the reperfusion of the spinal cord. These findings indicated that IRI may occur in the chronic severe compression of the spinal cords. It has previously been established that neurons in the spinal cord and subcellular components in the myelin sheath have biological membranes which are critical in sustaining normal physiological function and metabolism. Biological membranes contain a large amount of unsaturated fatty acids which are vulnerable to free radicals (37,39). Therefore, it could be proposed that susceptible membrane are attacked by overproductive free radicals brought by excessively reperfused blood, which leads to the deterioration of lipid peroxidation and may result in IRI. According to this hypothesis, potent antioxidants may be important in the management of spinal cord IRI. In clinical practice, the acute removal and decompression of the tumor may result in immediate cord expansion within the open canal space, and the long-term ischemic compressed segment of the cord is exposed to a rush in blood supply. This sudden cord expansion and reperfusion may have lead to disruption in the blood spinal cord barrier, and triggered a cascade of IRI resulting in postoperative neurologic deterioration.

It has been proposed that various pathogenic mechanisms including mitochondria-dependant apoptosis, inflammatory reactions, and specific phospholipid signaling cascades, may serve important roles in IRI (15,27–29,37,38). Chronic spinal cord ischemic injury may induce the passage of blood borne or neurotrophic substances (specifically TNF-α) through the blood brain barrier past its saturation point (40). It appears that decoupling of astrocyte foot processes from endothelial cell surfaces inhibits tight junction function in the blood brain barrier. Transport systems and ionic buffering would then be disrupted allowing worsened reperfusion injury upon decompression of a previously ischemic spinal cord. However, definite specific mechanisms for decompression-induced IRI have not yet been established and further study is required.

The present study highlights IRI may result from a delayed yet severe neurological deterioration in the absence of direct insult to the spinal cord following total removal of intraspinal meningiomas. Substantial efforts should be taken on the mitigation of spinal cord ischemic injury in clinical practice, including surgical techniques, pharmacological interventions, and mechanical methods. The present study may aid to improve the preoperative informed decision making process and further investigation into the underlying pathophysiological mechanisms of this ﬁnding are merited.

## Figures and Tables

**Table I.** Groups information in the study.

	Sham	Mild compression	Severe compression
Non-decompression	Sham group	MC group	SC group
Decompression	Sham-d group	MC-d group	SC-d group

d, decompression; MC, mild compression; SC, severe compression.
